# Engineered chirality of one-dimensional nanowires

**DOI:** 10.1126/sciadv.adx4761

**Published:** 2025-06-13

**Authors:** Megan Briggeman, Elliott Mansfield, Johannes Kombe, François Damanet, Hyungwoo Lee, Yuhe Tang, Muqing Yu, Sayanwita Biswas, Jianan Li, Mengchen Huang, Chang-Beom Eom, Patrick Irvin, Andrew J. Daley, Jeremy Levy

**Affiliations:** ^1^ Department of Physics and Astronomy, University of Pittsburgh, Pittsburgh, PA 15260, USA.; ^2^ Pittsburgh Quantum Institute, Pittsburgh, PA 15260, USA.; ^3^ Department of Physics, University of Strathclyde, Glasgow G1 1XQ, UK.; ^4^ Department of Physics, University of Liège, 4000 Liège, Belgium.; ^5^ Department of Materials Science and Engineering, University of Wisconsin-Madison, Madison, WI 53706, USA.; ^6^ Department of Physics, University of Oxford, Oxford, UK.

## Abstract

The origin and function of chirality in DNA, proteins, and other building blocks of life represent a central question in biology. Observations of spin polarization and magnetization associated with electron transport through chiral molecules, known collectively as the chiral induced spin selectivity effect, suggest that chirality improves electron transfer. Using reconfigurable nanoscale control over conductivity at the LaAlO_3_/SrTiO_3_ interface, we create chiral electron potentials that explicitly lack mirror symmetry. Quantum transport measurements on these chiral nanowires reveal enhanced electron pairing persisting to high magnetic fields (up to 18 tesla) and oscillatory transmission resonances as functions of both magnetic field and chemical potential. We interpret these resonances as arising from an engineered axial spin-orbit interaction within the chiral region. The ability to create one-dimensional electron waveguides with this specificity creates opportunities to test, via analog quantum simulation, theories about chirality and spin-polarized electron transport in one-dimensional geometries.

## Teaser

Artificial chirality in nanowires offers a controllable platform to study nature’s mysterious molecular handedness.

## INTRODUCTION

The relationship between chirality and electron transport has been investigated for two decades, following pioneering work by Naaman and Waldeck who first demonstrated a chiral induced spin selectivity (CISS) effect in photoemission experiments (*1*). It has been postulated that molecular chirality produces an axial spin-orbit interaction that locks the electron spin to its momentum, inhibiting back scattering and providing a potential rationale for the prevalence of chirality in biology (*2*). There have been numerous experiments linking molecular chirality to a variety of magnetic effects (*3*, *4*), and, recently, terahertz light pulses have been used to induce chirality in a non-chiral piezoelectric material (*5*). However, there are many open questions regarding the origin of CISS effects, including the importance of the helical radius, pitch, and spin-orbit interactions at the ends of these molecules. Experiments are usually performed at room temperature where it is difficult to disentangle the role of lattice dynamics.

**Fig. 1.**
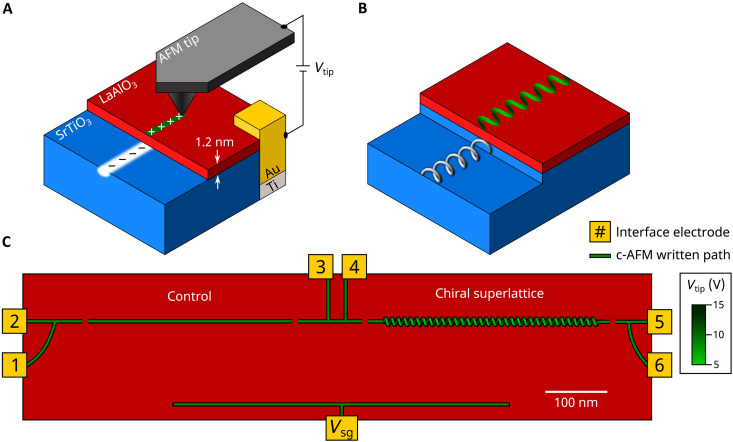
Fabrication and design of chiral superlattice devices at the LaAlO_3_/SrTiO_3_ interface. (**A**) Conductive atomic force microscopy (c-AFM) lithography is used to create conducting channels at the LaAlO_3_/SrTiO_3_ interface. (**B**) Schematic of a chiral conducting channel at the LaAlO_3_/SrTiO_3_ interface created via vertical and lateral modulations of the tip voltage and tip position respectively. (**C**) Schematic of device A. The device has two sections: a control waveguide (left) and chiral superlattice (right). Gaps in the nanowires denote tunnel barriers. Each section of the device can be probed independently with a four-terminal measurement, sourcing current through only the section of interest and measuring the voltage drop across that section. Current and voltage leads $\left(I_{+},I_{-},V_{+},V_{-}\right)$ are configured as (2, 4, 1, 3) for the control device and (5, 3, 6, 4) for the chiral device. Local permutations of these configurations do not affect the characteristic behavior of the devices.

One approach to understanding electron transport in chiral quasi–one-dimensional (quasi-1D) structures involves building and studying them in highly controllable systems, in the sense of analog quantum simulation (*6*, *7*). Quantum simulators based on ultracold atoms in optical potentials have replicated a wide range of phenomena affecting mesoscopic quantum transport (*8*–*11*), including creation of synthetic gauge fields, spin-orbit interactions (*12*, *13*), and quantum point contacts with quantized conductance (*14*). With advances in fabrication and control semiconductor quantum dot devices are another platform to offer versatility, great tunability, and simulational power (*15*, *16*). In our work, we make use of the extreme nanoscale programmability of the metal-insulator transition in LaAlO_3_/SrTiO_3_ heterostructures, where conductive nanoscale channels can be “sketched” by scanning a conductive atomic force microscopy (c-AFM) tip over the LaAlO_3_ surface. A positively biased AFM tip locally switches the LaAlO_3_/SrTiO_3_ interface from an insulating state to a conductive state (Fig. 1A), while a negatively biased AFM tip locally restores the insulating phase.

One of the remarkable observations for the LaAlO_3_/SrTiO_3_ system is the high degree of quantization, in contrast to the relatively low mobility μ ~ 10^3^ cm^2^/V·s found in two dimensions. The LaAlO_3_/SrTiO_3_ interface has been shown to exhibit electronic transport that is quantized at or near integer multiples of $e^{2}\;/\;h$ , where e is the electron charge and h is the Planck constant, consistent with highly ballistic transport and Landauer quantization.

The LaAlO_3_/SrTiO_3_ system has already been used to simulate two different types of potentials. One class of devices involves creating a Kronig-Penney–like superlattice, in which the strength of the confinement potential was modulated periodically (period λ = 10 nm) within a channel (*17*), creating a manifold of new subbands and fractional conductance plateaus. A second class of devices involves serpentine modulation of the electron potential, creating new spin-orbit interactions that shift the subband minima of the engineered effective electron waveguide (*18*). Here, we combine these two approaches to create quasi-1D potentials that break chiral symmetry within LaAlO_3_/SrTiO_3_ heterostructures (*19*, *20*). These effective chiral nanowires are created through a combination of two inequivalent perturbations of the “straight” quantum wire. The first perturbation involves creating a path for a serpentine potential, given by $y\left(x\right)=y_{0}+y_{k}\text{sin}\left(2\pi x/\lambda \right)$ , where $y_{0}$ , $y_{k}$ , and λ are parameters that can be programmed. The second perturbation involves performing c-AFM lithography with a sinusoidally varying tip voltage: $V_{\text{tip}}\left(x\right)=V_{0}+V_{k}\text{sin}\left(2\pi x/\lambda +\phi \right)$ . The expected impacts of the two combined perturbations on the local electron density are lateral and vertical displacements of the wave function, with the two displacements shifted by a phase ϕ . Choices of ϕ=±π/2 rad are expected to yield potentials that break mirror symmetry (Fig. 1B).

A schematic of one of the devices (device A) is shown in Fig. 1C. The device contains a “helix” potential structure on the right and a “control” potential structure on the left. The helix is created with $y_{k}$ = 10 nm, λ = 10 nm, and ϕ=π/2 . The control waveguide is straight and written with a constant tip voltage of $V_{\text{tip}}=12$ V. Each nanowire is bounded by two nanoscale junctions, created with negative voltage pulses $V_{\text{tip}}=-10$ V. A side gate, also created using c-AFM lithography, enables an applied voltage $V_{\text{sg}}$ to tune the chemical potential of both the helix and control device. The two electron waveguides can be independently characterized using four-terminal transport measurements, sharing now only the small section between leads 3 and 4. The tip position and voltage while writing a chiral superlattice device are shown in fig. S1.

## RESULTS

**Fig. 2.**
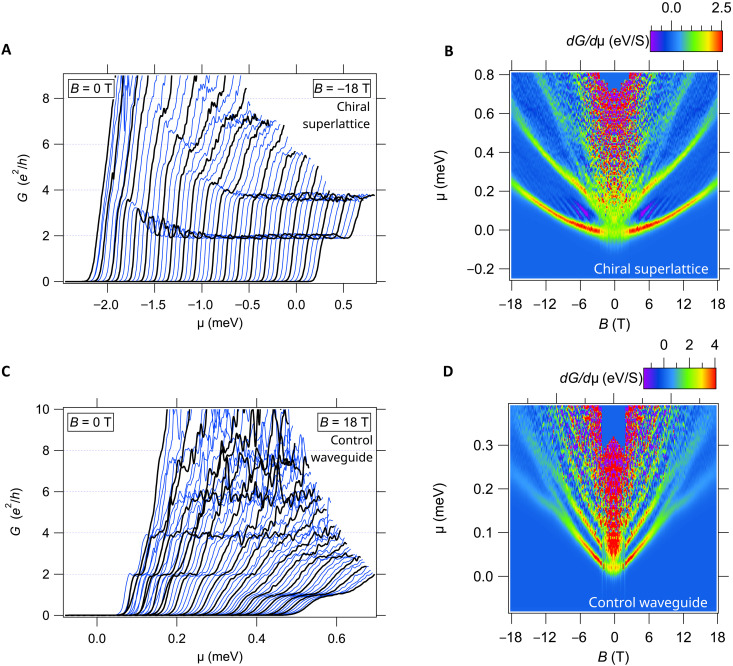
Device A transport data at *T* = 25 mK. (**A**) Conductance data for the chiral superlattice section of device A. Conductance *G* versus chemical potential μ, each curve is at a different applied magnetic field from *B* = 0 T to *B* = −18 T. Curves at 1-T intervals are highlighted in black. Curves are offset for clarity. (**B**) Transconductance dG/dμ as a function of magnetic field *B* and chemical potential μ. Bright (red/yellow) regions indicate increases in the conductance when new subbands become occupied. Light blue regions are zero transconductance and indicate conductance plateaus. Dark regions (purple/dark blue) are negative transconductance and indicate decreases in conductance. (**C**) Conductance data for the control waveguide section of the device. (**D**) Transconductance map of the control section of the waveguide. There are no oscillations observed in the control section of the device. It also has a lower pairing field of about *B* = 8 T, where the $2e^{2}\;/\;h$ plateau splits into steps of $1\;e^{2}\;/\;h$ . Transconductance data is symmetrized.

Figure 2A shows the four-terminal conductance of the chiral device A as a function of the chemical potential μ, for magnetic fields ranging between 0 and −18 T. (The choice of positive or negative magnetic fields is arbitrary, because the observed transport is symmetric with respect to magnetic field.) The chemical potential is given by $\mu =\mu_{0}+\alpha V_{\text{sg}}$ where α is calculated from finite-bias spectroscopy experiments (fig. S2F) (*21*), and $\mu_{0}$ is adjusted such that μ = 0 at the onset of the lowest electronic subband. Positive magnetic field values are shown in fig. S3, positive and negative magnetic field sweeps show similar transport. Each curve is taken at a different applied magnetic field *B* from *B* = 0 T on the left to *B* = −18 T on the right, with curves at 1 T intervals highlighted in black and offset for clarity. Conductance curves show plateaus at values close to quantized values $G=2e^{2}\;/\;h$ and $G=4e^{2}\;/\;h$ up to *B* = −18 T. At low values of magnetic field the $2e^{2}/h$ plateau is not visible, but there is a feature close to $4e^{2}\;/\;h$ . The transconductance dG/dμ for the chiral superlattice device (Fig. 2B) is calculated by numerical differentiation of the conductance curves in Fig. 2A. The bright red/yellow regions correspond to increases in conductance, when new subbands become occupied. The blue regions correspond to conductance plateaus. The purple regions correspond to regions of negative differential conductance.

**Fig. 3.**
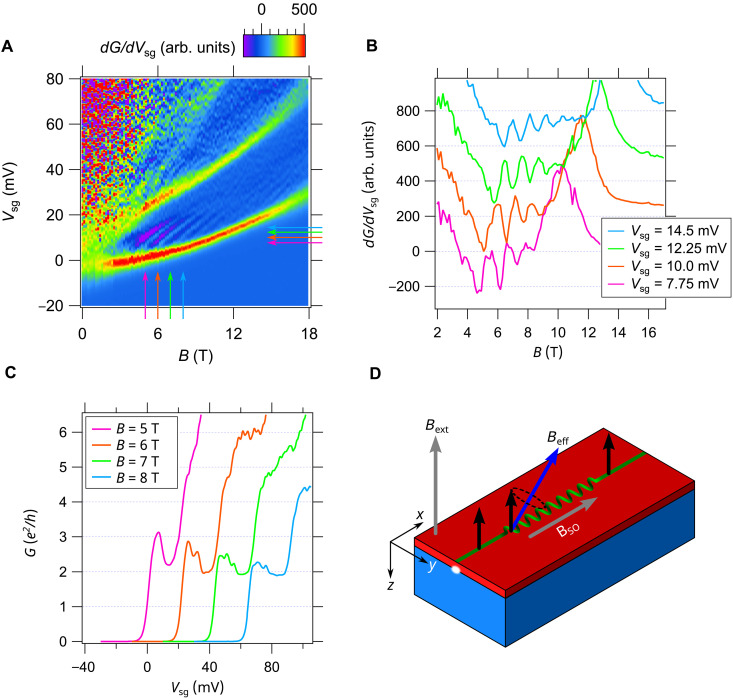
Characteristic oscillatory behavior in transport through chiral nanowires. (**A**) Transconductance dG/dμ showing oscillations in the region of the $2e^{2}\;/\;h$ plateau, the region between the lowest subband, and the next subband (bright red and yellow regions). (**B**) Horizontal line cuts of the transconductance data as a function of magnetic field *B*. Each curve is taken at increasing values of chemical potential μ, and curves are offset for clarity. (**C**) Vertical line cuts showing conductance values as a function of μ at different magnetic field values. The number of oscillations increases with increasing *B* field. The curves overshoot then reduce back to $2e^{2}\;/\;h$ . (**D**) Schematic of how the chiral superlattice device affects the spin of the electrons in the device. The applied external field and engineered spin orbit field from the superlattice create a new effective field inside the superlattice. In the straight portions of the nanowire, electrons are polarized in the *z* direction due to the external *B* field. When they enter the superlattice portion of the device that they precess around the effective field. When they exit the superlattice, they again will be polarized in the *z* direction. If they do not make a full precession by the time that they exit the superlattice, then the conductance will be suppressed giving rise to the oscillations.

The transconductance map reveals characteristic oscillations in the conductance, purple bands in the region above the lowest subband. Fainter sets of oscillations also exist for higher subbands and at much higher magnetic fields. Here, we focus on the strongest set of oscillations that are oriented at an “angle” in the μ-*B* plane and depend on both on the strength of the magnetic field and on the chemical potential. Line cuts of the transconductance and conductance highlighting these oscillations are shown in Fig. 3 (B and C). Vertical conductance line cuts show that the conductance initially rises above $G=2e^{2}\;/\;h$ and then falls back down to close to $2e^{2}\;/\;h$ . The number of oscillations increase as the magnetic field is increased. Figure 3C at *B* = 5 T shows one oscillation which increases to 2 oscillations at *B* = 6 T. The magnitude of the oscillations becomes suppressed at high magnetic field values, but they are still faintly visible, and, even at *B* = −18 T, the conductance still goes above then back down to $2e^{2}\;/\;h$ . The number of oscillations also increases with increasing values of μ shown in the horizontal line cuts of the transconductance map in Fig. 3B.

The control potential structure of device A (Fig. 2, C and D), an unmodulated straight effective electron waveguide connected in series with the chiral potential, behaves similarly to other published reports (*21*, *22*). The electron waveguide shows evidence for quantized electron pair transport with $G=2e^{2}\;/\;h$ at zero magnetic field up to a critical magnetic field *B_p_* = 8 T. Above that critical field, the paired state splits, and the lowest plateau becomes quantized at $G=e^{2}\;/\;h$ . Unlike the chiral device, there are no conductance oscillations found in the control device and no region of negative transconductance.

Finite-bias spectroscopy for the chiral potential (fig. S2A) shows the conductance map as a function of $V_{4T}$ and side gate $V_{\text{sg}}$ at *B* = 0 T and the corresponding superconducting peak in the conductance. Figure S2B shows a typical current-voltage relation (*I*-*V*) curve for the chiral superlattice section with a critical current of around 10 nA. *I*-*V* curves for the control device do not show a similar peak, but this could be due to the different back gating conditions for the two sections that were necessary to tune the device with the side gate.

Figure S2 (D and F) shows the finite bias spectroscopy for the conductance and transconductance of the chiral superlattice section, respectively. The transconductance map reveals characteristic diamonds (*23*, *24*), which are used to calculate the lever arm and convert gate voltage to chemical potential.

## DISCUSSION

We have explored several single-particle theoretical frameworks to explain our experimental observations of electron transport in chiral nanowires. These include Fabry-Pérot interference mechanisms, renormalized *g*-factor models, spin-orbit coupling (SOC) effects, and multi-orbital band degeneracies (see section 5 of the Supplementary Materials for detailed analysis). While each of these approaches captures certain aspects of the data, none provides a complete explanation for all observed conductance features, particularly the characteristic oscillations above the $2e^{2}\;/\;h$ plateau and the enhanced electron pairing at high magnetic fields. Our theoretical analysis indicates that a model combining effective attractive interactions with SOC between subbands naturally accounts for two key phenomena observed in our measurements: (i) enhanced electron pairing that persists to unusually high magnetic fields, and (ii) non-integer conductance oscillations that depend on both chemical potential and magnetic field strength.

### Enhanced electron pairing

The locking of the lowest electronic subbands into a $2e^{2}\;/\;h$ and $4e^{2}\;/\;h$ conductance plateau that persist to higher magnetic fields (*B* ~ 18 T) than for unmodulated devices indicates that the underlying pairing interactions between the electrons may be enhanced because of the chiral structure of the potential. The microscopic origin of electron-electron interactions in LaAlO_3_/SrTiO_3_ is still an active subject of research. As discussed in (*17*, *18*, *25*), electron pairing could be enhanced due to transverse spin-orbit interactions engineered by the potential modulations. However, these spin-orbit interactions can stabilize not only spin-singlet (*S* = 0) pairs but also spin-triplet (*S* = 1) pairs.

To study this effect, we generalized the mean-field theory developed in (*17*, *21*, *25*, *26*) (see section 3 of the Supplementary Materials for more details) to include both the chiral potential and transverse SOCs, using states characterized by transverse orbital and spin degrees of freedom as a single-particle basis. We find that effective attractive interactions lead to pairing in the lowest two subbands considered here and that this persists at higher fields for some chemical potentials when we add the chiral perturbation and the SOC. Moreover, our simulations show the coexistence of singlet and triplet pairs in the chiral region, where the triplet pairs are stabilized by the SOC induced by lateral modulations (*25*). An intuitive explanation for the enhanced pairing is that the SOC term facilitates the transfer of electrons between two distinct subbands. It favors delocalization of the electron over both bands, and, if these bands are close in energy, then attractive interactions will further lower the system’s energy by forming electron pairs (*25*).

**Fig. 4.**
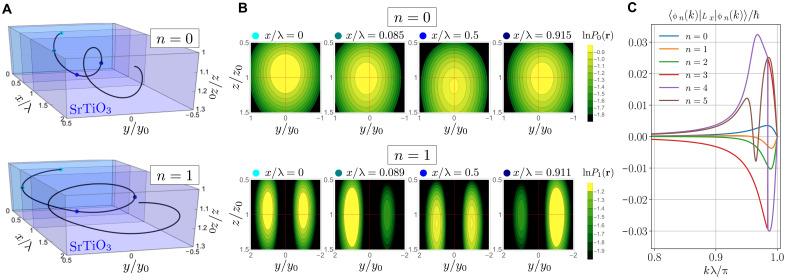
Theoretical model of electron probability density and orbital angular momentum in the chiral potential. (**A**) Trajectory of the center of mass of the electron probability density along the waveguide in the first (*n* = 0, top), and second (*n* = 1, bottom) eigenstate $|\;\phi_{n}\left(k\right)\;\rangle$ of the trapping potential for a quasimomentum k=0.99π/λ . (**B**) 2D slices of the previous panel showing the natural logarithm of the normalized probability density $P_{n}\left(r\right)={|\;\langle r|\phi_{n}\left(k\right)\rangle \;|}^{2}$ in the transverse plane at different positions *x* for *n* = 0 (top) and *n* = 1 (bottom). For both cases, we observe a precession of the probability density. Here, $y_{0}=\sqrt{\hbar \;/\;\left(m_{y}\omega_{y}\right)}$ is the characteristic size of the harmonic oscillator in the lateral (*y*) direction (and, correspondingly, *z*_0_). (**C**) Expectation value of the axial orbital angular momentum for the lowest lying energy eigenstates $\langle \phi_{n}\left(k\right)|\;L_{x}\;|\phi_{n}\left(k\right)\rangle$ as a function of quasimomentum near the Brillouin zone edge. Parameters of the numerical simulations are $m_{x}=m_{y}=1.9m_{e}$ , $m_{z}=6.5m_{e}$ , A=λ=10 nm, δ = 0.5, $y_{0}=26$ nm, and $z_{0}=8$ nm.

To understand the physical effect of the c-AFM tip modulations on the structure of the single-particle eigenstates in the waveguide, we introduce a chiral harmonic oscillator model which explicitly breaks chiral symmetry (see section 2 of the Supplementary Materials). The effective single-particle physics is captured in Fig. 4, which shows the trajectory of the center of mass (A) and the probability density of the lowest eigenstates (B) in the waveguide as well as their respective axial orbital angular momentum (C). We find eigenstates with nonzero orbital angular momentum $\langle L_{x}\rangle \neq 0$ (see section 2 of the Supplementary Materials alongside the provided video). Inside the perturbed region, the electron pairs should thus carry finite $L_{x}$ , with their spin degree of freedom generally distributed over the singlet and triplet sectors.

### Conductance oscillations

The conductance oscillations shown in Fig. 2 take on non-integer values of $e^{2}\;/\;h$ above $2e^{2}\;/\;h$ , which suggest that they arise because of a scattering process for pairs of electrons passing through the waveguide. To account for this behavior, we first note that the modulations of the confining potential give rise to center-of-mass orbits similar to those of a charged particle in a magnetic field, which are expected to produce an effective axial magnetic field $B_{\text{SO}}$ that arises from an axial SOC (*27*). Figure 3D shows a schematic of how an engineered axial magnetic field in a finite-length modulated segment, surrounded by unmodulated sections, can produce oscillations in the transport as a function of an applied out-of-plane magnetic field. The effective axial magnetic field, combined with an applied external magnetic field in the *z* direction, changes the spin quantization axis for the charged particles traveling within the chiral waveguide. Particles in the unmodulated portion of the device will be spin polarized in the direction of the applied external magnetic field. As they enter the chiral waveguide, a Rashba-like spin-orbit interaction should cause coherent spin precession around the new quantization axis of the effective *B* field. The finite length of the device will cause transmission resonances that are periodic in the number of complete precessions. The nature of the oscillations should change depending on the strength of the applied *B* field and the energy of the particles determined by the chemical potential.

This analysis provides an underlying explanation for the observed conductance oscillations and can be captured in a phenomenological scattering model describing transmission resonances with similar oscillations to those observed in the transconductance data shown in Fig. 2. As the pairs propagate through the waveguide as described above and shown in Fig. 4, a spin-orbit interaction of the form $\vec{L}\cdot \vec{S}$ drives coherent oscillations between the singlet and triplet states. This interaction is absent for the lowest paired state, whose evolution is, therefore, confined to the singlet sector throughout the waveguide, giving rise to a stable $2e^{2}\;/\;h$ conductance plateau. We model the singlet-triplet interaction for the first excited paired state by an effective scattering problem of a pseudo spin-^1^/_2_ particle. The singlet-triplet oscillations manifest as an oscillating conductance, with an amplitude of at most $2e^{2}\;/\;h$ , because only the singlet pairs are stable in the lead and triplets cannot be observed. The oscillating conductance from the first excited state contributes on top of the stable ground-state baseline of $2e^{2}\;/\;h$ . We fit the resulting transmission probability to the transconductance fringes (see section 4 of the Supplementary Materials) and find good agreement with the experimental data in Fig. 2.

Programmatic control of chirality in LaAlO_3_/SrTiO_3_ effective electron waveguides significantly expands the capabilities of this 1D analog quantum simulation platform, enabling the simulation of chiral structures that are believed to be important for spin selectivity in biological systems, donor-acceptor molecules (*28*), the role of coherence (*29*), and other manifestations of the CISS effect. Unlike most naturally occurring systems, the LaAlO_3_/SrTiO_3_ platform offers a range of mesoscopic building blocks to model not just chirality but also polarity at the endpoints, which is also believed to contribute to CISS. Future experimental directions, involving segments with varying pitch or opposing chirality, measurements with a combination of out-of-plane and in-plane applied magnetic fields, and in highly nonequilibrium conditions, will provide further insights into the interrelationships between electron transport and chirality. While the microscopic origin of electron pairing in LaAlO_3_/SrTiO_3_ and aspects of observed ballistic transport are still not well understood, these are well described by phenomenological models. Further investigation supported by the use of chiral transport will help to lower the “intellectual entropy” that gives rise to uncertainties in the models used to describe these programmed quantum systems, which will enable chiral 1D systems to serve as building blocks to form 2D superlattices with interesting engineered and/or topological spin textures.

## MATERIALS AND METHODS

LaAlO_3_/SrTiO_3_ samples (3.4 unit cells) were grown using pulsed laser deposition described in more detail elsewhere (*30*). Electrical contact was made to the interface by ion milling and depositing Ti/Au electrodes. c-AFM writing was performed by applying a voltage bias between the AFM tip and the interface, with a 1-gigaohm resistor in series. c-AFM writing was performed at room temperature in 30 to 40% relative humidity using an asylum MFP3D AFM. Written samples were then transferred into a dilution refrigerator and cooled to a base temperature of 25 mK. Magnetic fields up to 18 T were applied in an out-of-plane geometry. Four-terminal measurements were performed using standard lock-in techniques at a reference frequency of 13 Hz, with an applied ac voltage of 100 μV. *I*-*V* curves are also measured by applying a finite dc bias to the device.

The chiral superlattice section of the device (right) is written with a sinusoidal modulation of the tip voltage ( $V_{0}=10$ V, $V_{k}=5$ V, and $\phi =90^{\circ }$ ) and a lateral modulation amplitude ( $y_{k}=5$ nm and 2π/k=10 nm), with 34 total periods. The superlattice is surrounded by two straight segments written with $V_{\text{tip}}=12$ V, in which highly transparent tunnel barriers (*21*) are created by applying negative voltage pulses $V_{\text{tip}}=-10$ V to the tip while writing.
