# Supplementary material for: Engineered chirality of one-dimensional nanowires
Source: Sci Adv. 2025 Jun 13;11(24):eadx4761. doi: 10.1126/sciadv.adx4761 (PMC12164949; doi:10.1126/sciadv.adx4761)
Supplement: Supplementary file 1 — Supplementary Text Figs. S1 to S9 Legend for movie S1 [file sciadv.adx4761_sm.pdf]

Science Advances
AAAS

Supplementary Materials for

Engineered chirality of one-dimensional nanowires

Megan Briggeman *et al.*

Corresponding author: Jeremy Levy, jlevy@pitt.edu

Sci. Adv. **11**, eadx4761 (2025)
DOI: 10.1126/sciadv.adx4761

The PDF file includes:

Supplementary Text
Figs. S1 to S9
Legend for movie S1

Other Supplementary Material for this manuscript includes the following:

Movie S1

1 Secondary Device

A second Device B (Fig. S4), shows qualitatively similar features. The chiral potential of this device was written with the same serpentine path parameters, but the vertical modulation was first written with a constant tip voltage $V_{\rm tip} = 10$ V, and then the path was rewritten with the modulated voltage $V_{\rm tip} = \pm 2.5$ V. This two-pass writing process is closer to the way in which the electron waveguide devices are created (22). This device also shows oscillations in conductance above the $G = 2\ e^2/h$ plateau (Fig. S4D). Additionally, there are some periodic features in the we^2/h plateau which are visible in Fig. S4C. In the conductance these features are due to a periodic change in the slope of the conductance jump from an insulating state to the $2\ e^2/h$ plateau.

2 Waveguide models

Here we dicuss the structure of the eigenstates of the electrons in the waveguide in more detail. We present two distinct waveguide models that capture the chiral perturbation and yield qualitatively similar single-particles eigenstates and orbital momentum features. The first (more accurate) model accounts for a trapping potential along z that takes into account the fact that the electrons cannot penetrate in the $LaAlO_3$ layer (half-harmonic potential with spatially-dependent frequency). The second model consists in a simplified version where the confinement along z is a full-harmonic potential with a modulated center.

2.1 Chiral model

Consider a particle propagating along the axial (x) direction, and confined transversally by a laterally and vertically modulated harmonic oscillator potential. The Hamiltonian reads

$$H = \frac{p_x^2}{2m_x} + \frac{p_y^2}{2m_y} + \frac{1}{2} m_y \omega_y^2 \left(y - A \sin[2\pi x/\lambda]\right)^2 + \frac{p_z^2}{2m_z} + \frac{1}{2} m_z \omega_z^2 (1 + \delta \cos[2\pi x/\lambda])^2 z^2 , \quad z \geq 0 \tag{S1}$$

where λ is the wavelength of the modulation, A is shift in the centre of the lateral harmonic oscillator potential, and $\omega_{y,z}$ the trapping frequencies in the transverse (y and z) directions. We emphasize that we are treating the confinement along z as a modulated half-harmonic oscilltor potential (which

is infinite for $z < 0$). These chiral modulations lead to an effective coupling between the transverse degrees of freedom, such that the Bloch eigenfunctions $|\phi_n(k)\rangle$ will be superpositions of different occupations in the two transverse directions $|k, m_y, n_z\rangle$.

Fig. 4 (A-B) shows the probability densities of the ground, and first excited state near the Brillouin zone edge, $|\phi_{0,1}(k\lambda = 0.99\pi)\rangle$. For both states, the centre of mass of the probability density $P_n(\mathbf{r}) = |\langle \mathbf{r}|\phi_n(k)\rangle|^2$ traces a helical path along the axial direction. The helical undulation means that the eigenstates carry finite axial orbital angular momentum close to the Brillouin zone boundary, $\langle L_x \rangle$, as shown in panel (C).

In our model, these eigenstates constitute the relevant conductance channels in the patterned $LaAlO_3/SrTiO_3$ waveguide (with an additional spin degree of freedom omitted here for simplicity). Interactions (see the discussion in the main text) will lead to pairing, resulting in $2e^2/h$ of conductance from each paired transport channel, and are modelled below as attractive interactions in a mean-field theory.

2.2 Helical model

We also investigated the following simplified waveguide Hamiltonian

$$H = \frac{p_x^2}{2m_x} + \frac{p_y^2}{2m_y} + \frac{1}{2}m_y\omega_y^2\,(y - A\sin[2\pi x/\lambda])^2 + \frac{p_z^2}{2m_z} + \frac{1}{2}m_z\omega_z^2\,(z - A\cos[2\pi x/\lambda])^2 \ , \quad \text{(S2)}$$

where the vertical waveguide perturbation was modelled as a direct shift of the vertical harmonic potential. This model yields a true helical motion of the electron probability density and, as for the previous model, a non-zero orbital momentum along x (not shown). We thus conclude that the exact form of the microscopic model for the waveguide is not important: it is the breaking of the chiral symmetry in both models that yields this universal behaviour.

3 Hartree-Fock-Bogoliubov Theory

To study the effect of interactions in the modulated waveguide we developed a self-consistent Hartree-Fock-Bogoliubov theory (17, 21, 25, 26) in the presence of periodic potentials (which we note in 1D will not describe superconductivity but will properly predict pairing, which is our focus here). We neglect the weak potential along the axial direction in this minimal model, and

treat the electrons as free particles along x. The single-particle Hamiltonian in the Landau gauge $\mathbf{A} = (-By, 0, 0)^T$ reads

$$H = \sum_{i=\{x,y,z\}} \frac{\Pi_i^2}{2m_i} + \frac{1}{2} m_y \omega_y (y - y_x)^2 + V(z) + A_z \cos(Qx) - \frac{1}{2} g \mu_B B \sigma_z - \mu + \frac{\alpha_v \sigma_y - \alpha_l \sigma_z}{\hbar} \Pi_x \ . \quad \text{(S3)}$$

The first three terms describe the spin-degenerate kinetic energy of the electron with canonical momentum operators $\vec{\Pi} = \vec{p} - q\vec{A}$, and effective masses m_i. $V(z) = \frac{1}{2} m_z \omega_z^2 z^2$ for $z \geq 0$ (else ∞) is the half-harmonic oscillator confinement (see also discussion above), and $\omega_{y,z}$ the transverse harmonic oscillator frequencies. The vertical modulation of the waveguide is modelled by a Kronig-Penney potential $A_z \cos(Qx)$, whilst the lateral modulation shifts the centre of the harmonic oscillator in the lateral (y) direction, $y_x = A_y \sin(Qx)$. The next terms describe the lifting of the spin degeneracy due to an external magnetic field B via the Zeeman term, the chemical potential μ, and finally a static Rashba spin-orbit coupling (SOC) (25). H_z is a half-harmonic oscillator whose eigenstates are the odd regular harmonic oscillator states, i.e. $E_z = \hbar\omega_z(2n + 3/2)$ and $\psi(x, y, z) = \psi(x, y)\psi_n(z)$ since $[H, H_z] = 0$. The vertical degree of freedom in this model is fully decoupled from the lateral and axial direction (i.e. $[H, H_z] = 0$ while $[H_x, H_y] \neq 0$), but for simplicity we will neglect axial-lateral correlations, and assume the eigenfunctions to be separable in the different directions. As we shall see in the following section this assumption neglects structures in the transverse degrees of freedom, but here we are mainly interested in modelling the effect of electron pairing.

The resulting single-particle Hamiltonian is periodic in x. Its eigenfunctions are Bloch waves along the axial directions, and (half) harmonic oscillator eigenstates in the (vertical) lateral directions. Expanding the wavefunction in a plane wave axial basis, $|\psi\rangle = \sum_{n_y,n_z,\sigma} \sum_{q,G} \psi^{(G)}_{n_y,n_z,q,\sigma} |q + G, n_y, n_z, \sigma\rangle$, where we have restricted the quasimomentum q to the first Brillouin zone, and introduced the reciprocal lattice vector $G = \pm Q, \pm 2Q, \ldots$ with $Q = 2\pi/\lambda$, the eigenproblem $H|\psi\rangle = \mu|\psi\rangle$ then takes the compact form

$$\begin{aligned} \sum_{G'} \mathbf{\Psi}_q^{(G)\dagger} \Big[& \mathbb{Q}_{-2}\, \delta_{G',G+2Q} + \mathbb{Q}_{-1} \left(q + G + \frac{Q}{2} \right) \delta_{G',G+Q} + \mathbb{E}_q^{(G)}\, \delta_{G',G} \\ & + \mathbb{Q}_{+1} \left(q + G - \frac{Q}{2} \right) \delta_{G',G-Q} + \mathbb{Q}_{+2}\, \delta_{G',G-2Q} \Big] \mathbf{\Psi}_q^{(G')} = 0 \ , \end{aligned} \quad \text{(S4)}$$

where we have restricted ourselves to the lowest two subbands, $n_y = n_z = 0$, and $\mathbf{\Psi}_q^{(G)} =$

$(\psi^{(G)}_{0,0,q,\uparrow}\ \psi^{(G)}_{0,0,q,\downarrow})^T$, and thus neglected subband mixing as in (25), where it was shown to be valid for reasonable waveguide parameters. The $2x2$ block matrices read

$$\mathbb{E}^{(G)}_q = \left(\frac{\hbar\Omega}{2} + \frac{3\hbar\omega_z}{2} - \mu\right) - \frac{1}{2} g\mu_b B\sigma_z + \frac{1}{2} m_y \left(\frac{\omega_y\omega_c}{\Omega}\right)^2 \left(y^2_{q+G} + \frac{A^2_y}{2}\right) \\ + \ (\alpha_v\sigma_y - \alpha_l\sigma_z)\left(\frac{\omega_y}{\Omega}\right)^2 (q+G) \tag{S5}$$

$$\mathbb{Q}_{\pm1}(k) = \frac{A_z}{2} \pm \frac{i}{2} m_y \left(\frac{\omega_y\omega_c}{\Omega}\right)^2 y_k A_y \ \pm\ \frac{i}{2}(\alpha_v\sigma_y - \alpha_l\sigma_z) A_y \left(\frac{eB}{\hbar}\right)\left(\frac{\omega_y}{\Omega}\right)^2 \tag{S6}$$

$$\mathbb{Q}_{\pm2} = -\frac{1}{8} m_y \left(\frac{\omega_y\omega_c}{\Omega}\right)^2 A^2_y\ , \tag{S7}$$

where $y_k = \frac{\hbar k}{eB}$, $\Omega = \sqrt{\omega_y^2 + \omega_c^2}$ the renormalized trapping frequency along y, and $\omega_c = eB/\sqrt{m_x m_y}$ the cyclotron frequency. To write down a many-particle theory we promote the different Fourier amplitudes to fermionic operators in the usual way, and phenomenologically include attractive interactions to generate pairing at the mean-field level (17, 21, 25, 26). The interaction is parameterised by $U \equiv U(B) = U_0\sqrt{1 - \frac{\omega_c^2}{\Omega^2}} = U_0\frac{\omega_y}{\Omega}$, and has a phenomenological magnetic field dependence (25), where U_0 is a bare interaction strength. This makes $|U|$ a decreasing function of the magnetic field, and the mean-fields independent of this effective scaling. The interacting eigenvalue problem is then solved self-consistently. The eigenvalues and eigenvectors define quasiparticle operators, which are used to update the mean fields at each iteration step until convergence is reached (unless otherwise stated we run the self-consistent convergence procedure until the mean-fields are converged to below 1% relative precision.

Fig. S5 shows the pairing correlations of the mean-field model for the chiral waveguide, and control device (straight). Panel (a) shows the singlet pairing field Δ_s as a function of μ and B, where attractive interactions lead to singlet pairing in the two subbands up to some critical field B_{P}. The inclusion of the chiral perturbation and SOC in panel (b) reshapes, and increases the singlet pairing phase in the (B, μ) space to lower chemical potential values, and up to stronger critical magnetic fields for some chemical potentials. These findings are consistent with recent studies investigating separately the effect of laterally, and vertically induced SOC (17, 25). Note that for control straight waveguides, such a mean-field analysis has been shown to yield qualitative similar results to DMRG calculations (22).

Additionally, we expect the periodic modulation to open band gaps in the single-particle spectrum, which manifest as a small unpaired (zero-conductance) region in (B, μ) space, subdividing

the singlet pairing region into two. Qualitatively similar features, a fracturing of the transconductance lines due to a Kronig-Penney modulation, have been observed in (17). The bottom row shows the triplet pairing field Δ_t for the control device (c), and chiral waveguide (d). As shown in (25), laterally induced SOC stabilises triplet pairing, leading to the coexistence of stable singlet and triplet pairs in the chiral waveguide with $\Delta_{s/t} \gtrsim k_B T$.

4 Scattering Model

Here we propose a phenomenological scattering model to account for oscillations in the transmission probability, as the electron pairs propagate through the patterned $LaAlO_3/SrTiO_3$ waveguide.

Our interacting simulations showed that spin-orbit coupling can be induced by the chiral modulation and stabilises triplet pairs inside the chiral region, whereas only singlet states are present in the leads. A conductance measurement would therefore only measure the transmitted singlets, $P^t_{S=0}$. We expect this quantity to oscillate both as a function of external magnetic field B and particle energy E (tuned by the external chemical potential μ), and for simplicity we assume a perfect effective backscattering of the triplet pairs at the waveguide-lead boundary. We formulate the scattering problem in terms of a pseudo spin-$1/2$ particle (representing the singlet and triplet states of the pairs), entering the waveguide from the left lead, and being transmitted into the right lead with a probability P^t_S, with $S = \{0, 1\}$ for singlet/triplet respectively. The chiral waveguide is modelled as an axial spin-orbit coupling interaction for the particle which causes the spin and momentum to lock, and the particle to be subject to an effective axial magnetic field (which implicitly depends on its motional state). Since we hope to gain a qualitative understanding, we are not concerned with quantitative differences in the quantum numbers of a single particle or composite bosonic pairs.

To be concrete, consider two leads, $H_{\text{lead}} = \frac{p_x^2}{2m_x} + E_z\sigma_z$, coupled to a spin-orbit region of length L with Hamiltonian $H_{\text{sys}} = \frac{p_x^2}{2m_x} + \frac{\alpha}{\hbar} p_x\sigma_x + E_z\sigma_z$, where α denotes the strength of the axial spin-orbit coupling term, and $E_z = g\mu_B B/2$ the Zeeman energy.

We derive four boundary conditions per spin degree of freedom from the continuity of the wavefunction, and the probability flux at both interfaces $x = 0$ and $x = L$. Note that the probability flux is related to the particle velocity operator $v_x = \frac{i}{\hbar}[H, x] = \frac{p_x}{m_x} + \frac{\alpha}{\hbar}\sigma_x$ inside the central region (and $\alpha = 0$ in the leads). The scattering amplitudes of the leads are then implicitly related through

these boundary conditions, and are rewritten into a scattering problem of incoming and outgoing amplitudes, $\vec{O} = S\vec{I}$, related by the scattering matrix S. For a given input vector of scattering amplitudes $\vec{I}$, the outgoing amplitudes can now be computed straightforwardly, and reflection and transmission probabilites derived from the probability flux $j_\sigma(x) = \frac{i\hbar}{2m_x}\left[\psi^*_\sigma(x)\partial_x\psi_\sigma(x) - \psi_\sigma(x)\partial_x\psi^*_\sigma(x)\right]$.

The analytical solution to the scattering problem displays oscillations in both the magnetic field, and chemical potential. A first order perturbative expansion of the transmission probability in $E_{\rm so}/E_z$ (leaving the plane wave factors intact) yields the closed form expression

$$P^t = 1 - \left[(\zeta+\eta)\sin^2\left(\frac{k_+ + k_-}{2}L\right) + (\zeta-\eta)\sin^2\left(\frac{k_+ - k_-}{2}L\right)\right]\frac{E_{\rm so}}{E_z} + O\left[\frac{E_{\rm so}^2}{E_z^2}\right], \tag{S8}$$

where the momenta of the eigenstates in the spin-orbit coupled region are given by $(\hbar k_\pm)^2 = 2m_x\left[E + E_{\rm so} \mp \sqrt{(E+E_{\rm so})^2 + (E_z^2 - E^2)}\right]$, $E_{\rm so} = m_x\alpha^2/\hbar^2$ is the spin-orbit coupling energy, and L the size of the waveguide. The prefactors are given by $\zeta = [\xi_\downarrow^2 - 2 + (\xi_\downarrow^2 - 1)\xi_\uparrow^2]/(2\xi_\downarrow\xi_\uparrow)$ and $\eta = -(\xi_\downarrow^2 + \xi_\uparrow^2)/2$, where $\xi_\sigma = k_\sigma L_B, \hbar k_\sigma = \sqrt{2m_x(E \mp E_z)}$ are the momenta for the spin-polarised states in the leads, and $L_B = \sqrt{\hbar^2/(m_x E_z)}$ the magnetic length scale in the system. Since the transmission probability is related to the experimental conductance, we fit dP^t/dE to the transconductance fringes, and find consistent qualitative agreement with oscillation periods of $\Delta B \sim 1$T across a range of chemical potentials. We find that $\alpha = 0.45$ meV nm, and $g = 0.85$ provide the best estimates for the scattering model parameters, consistent with other studies on $LaAlO_3/SrTiO_3$ samples (25, 31). We note that there is a functional relationship between $\alpha \lesssim 2$meV nm and $g \lesssim 2$ which will give rise to similar paterns of maxima and minima in the transmission as these parameters are increased or decreased, which would also be consistent with the transconductance data. For larger $\alpha \gtrsim 2$ meV nm and $g \gtrsim 2$, additional oscillations appear along the fringes, which do not appear in the experimental data.

5 Alternative models we considered (and discounted)

In this section, we discuss alternative models we considered to explain the experimental data and why we discounted them.

5.1 Farby-Perot interference

A question one could naturally ask when looking at the oscillations of the conductance or transconductance data is whether the observed behaviour could be simply explained by the scattering of the particles due to a potential barrier without the need to include spin-orbit coupling as presented in the scattering model section above, nor attractive interactions to explain pairing of particles. This would produce Fabry-Perot-like interferences and oscillations in the transmission. However, this interpretation has two main shortcomings. First, the chiral potential is the combination of a vertical and lateral potentials, which have been investigated individually in previous works (17,18). If one accepts the assumption that the chiral potential can be viewed as a potential barrier associated with Fabry-Perot-like interferences, it would be reasonable to consider the same for the vertical and lateral potentials. However, none of them were producing conductance or transconductance oscillations. Second, the observed conductance oscillations exhibit an amplitude of more than e^2/h. The most reasonable explanation for such conductance are either the presence of spin-orbit coupling or attractive interactions leading pairs of particles. However, again, attractive interactions alone would not explain why conductance oscillations are not observed in the vertical or lateral potential experiments. Hence, we conclude that the most natural way to undertand the experimental data is to consider that the combination of vertical and lateral modulations leads not only to the effects observed in the individual experiments but also to an additional effect, namely a longitudinal spin-orbit coupling which, together with attractive interactions, yield conductance oscillations of more than e^2/h.

5.2 Renormalised g-factor

We investigated other possible explanations for the observed subband collapse and re-entrant 'pairing' in the data, without invoking an effective pairing interaction in the system. To this end we explored if a renormalisation of the g-factor of the system within a single-particle model could give rise to qualitatively similar features as the ones observed in previous experiments (21,22). We focus here on two particularly striking features in the transconductance data - (1) the coalescence of two subbands of opposite spin (but same orbital quantum numbers) below some critical magnetic field B_p, above which we observe a typical Zeeman splitting, and (2) the extended/collapsed (avoided)

crossings between subbands of different (same) spin and differing orbital states. Our starting point is a single-particle model which has been shown to capture qualitatively the subbands in these oxide heterostructures very well (21, 25). To model the 'pairing' features within this single-particle model we considered a variety of functional forms of the g-factor, and found the best agreement using an anomalous, magnetic field dependence of the form $g = g_{\text{eff}} \max\{0, 1 - B_p/B\}$ (26). Here B_p takes the role of a critical magnetic field beyond which the Zeeman term becomes dominant, and leads to a splitting of subbands while $g(B) = 0$ mimicks the observed subband collapse.

Fig. S7 shows the conductance map (a) as a function of both chemical potential and external magnetic field (the different subbands are indicated in red, and the functional form of $g(B)$ shown as a white dashed lines), and three linecuts (b) for $g_{\text{eff}} = 0.2$ and $B_p = 1T$. At low magnetic fields subbands differing only in their spin quantum number indeed are collapsed onto each other and give the impression of attractive pairing. For $B > B_p$ we see that the subbands split, as expected when the Zeeman term becomes the dominant contribution in the Hamiltonian. In this way this simple model seems to be able to capture feature (1) reasonably well. For feature (2) we turn our attention to the crossing of subbands $|0, 1, \downarrow\rangle$ with $|1, 0, \uparrow\rangle$ at $B \approx 5T$. In previous experiments these bands exhibited re-entrant pairing (21, 22), while an avoided crossing was observed between subbands such as $|0, 1, \downarrow\rangle$ with $|1, 0, \downarrow\rangle$. This is completely absent in this case, we do not observe any effect on a subband due to the presence of an energetically nearby subband. This is intuitively clear by virtue of the fact that this is a single-particle model. Furthermore, 'pairing' was observed experimentally between different subbands at different magnetic fields, which cannot be accounted for within this model because the $g(B)$ affects all subbands equally. In principle one could refine this model further by introducing an orbital dependence into the g-factor but we find this difficult to justify physically, and we concluded that interactions are indeed the most natural way to account for the observed subband collapse, and re-entrant pairing.

In a similar way multi-orbital or band degeneracies could in principle give rise to a subband collapse as observed experimentally in the Pascal conductance series (22). However, such degenracies require a carefully fine-tuned relation between the vertical and lateral confinements l_y and l_z, and we have no indication that this confinement relation is satisfied. Also, we can exclude spin degeneracy due to the strong external magnetic fields involved in the experiment.

5.3 Effect of spin-orbit coupling

We also investigated the effect of spin-orbit coupling (SOC) on the transport as a potential alternative explanation for the observed features in the experiment. We follow the discussion in (25) by introducing a single-particle model and including two forms of SOC; *lateral* SOC $\sim \alpha_l \sigma_z$ and *vertical* SOC $\sim \alpha_v \sigma_y$. Analogously to the previous section we compute the conductance G as a function of chemical potential and external magnetic field, shown in Fig. S9 and Fig. S8 for $\alpha_l = 2$ meV·nm and $\alpha_v = 3$ meV·nm respectively.

A lateral SOC does not appear to significantly affect the subband structure, and hence the transport properties. In (25) it was shown that this type of SOC can stabilise triplet pairing in a system with attractive interactions, but no change in the transport properties at the single-particle level was found, in agreement with our modelling here.

Fig. S8 in turn shows the affect of vertical SOC on the transport properties. We can identify very clear deviations from the subband structure in the absence of SOC. Notably the vertical SOC appears to open regions in (μ, B) parameter space where the conductance jumps up by $2e^2/h$ (and subsequently down by $1e^2/h$) with increasing chemical potential μ. Whilst we do observe oscillations of the conducatance with an amplitude of $\sim 2e^2/h$ it is important to highlight that these oscillations both increase and decrease G in the same way. However, in this model the increase and decrease of G are biased and *not* the same. In stark contrast to the experimental findings we do not observe oscillations of G around a stable conductance plateau with an amplitude of $\sim 2e^2/h$. We therefore conclude that this model also does not provide a comprehensive explanation for the observed features, and rather that interations are the most natural and simple explanation which can explain the different observed elements in the transport measurements.

6 Scattering Length Estimation

Here we estimate the scattering lengths for the control and helical devices near the $G = 2e^2/h$ plateau, following the method described in Ref. (21). In that method, we assume a scattering length $L_x = 2e^2/h(1 - \exp[-l_x/L_x])$, where $x = c$ denotes the control device and $x = h$ denotes the helical device. We find:

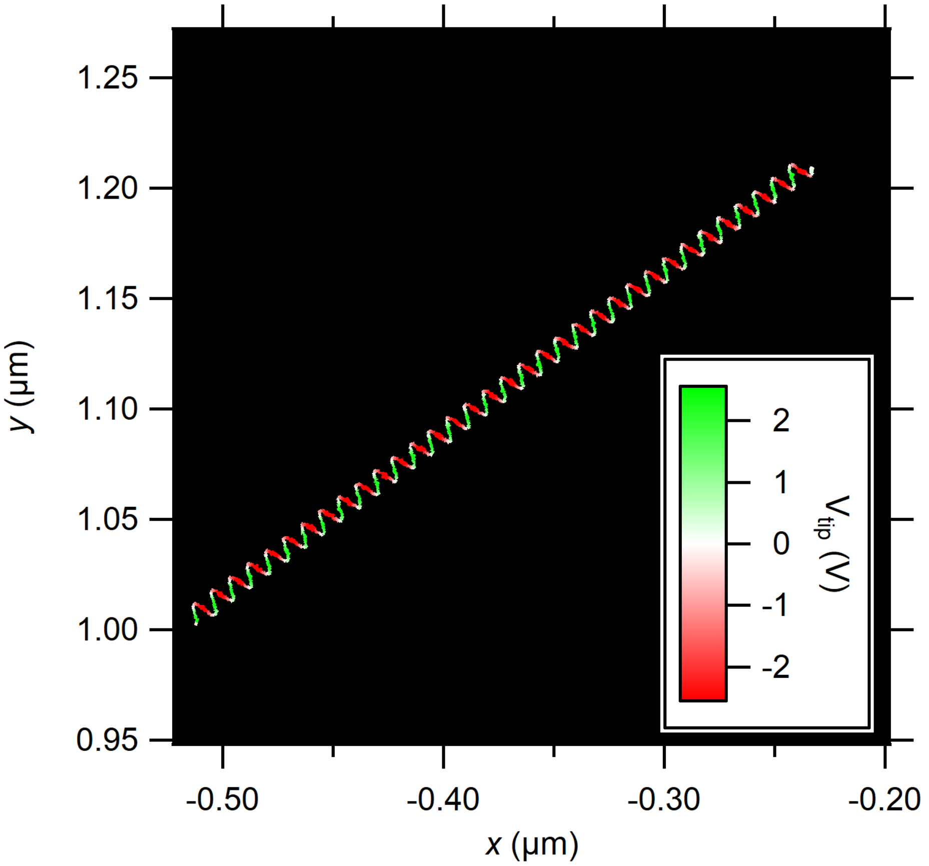

Figure S1: **Conductive AFM tip parameters used during fabrication of the chiral superlattice device.** AFM tip position and voltage monitored while writing a chiral superlattice section of Device B. Tip voltage V_{tip} is represented by the color of the curve.

Table S1: Estimated scattering lengths for control and helical device near $G = 2e^2/h$ plateau.

L_c	L_h
14±4 μm	8 ± 3 μm

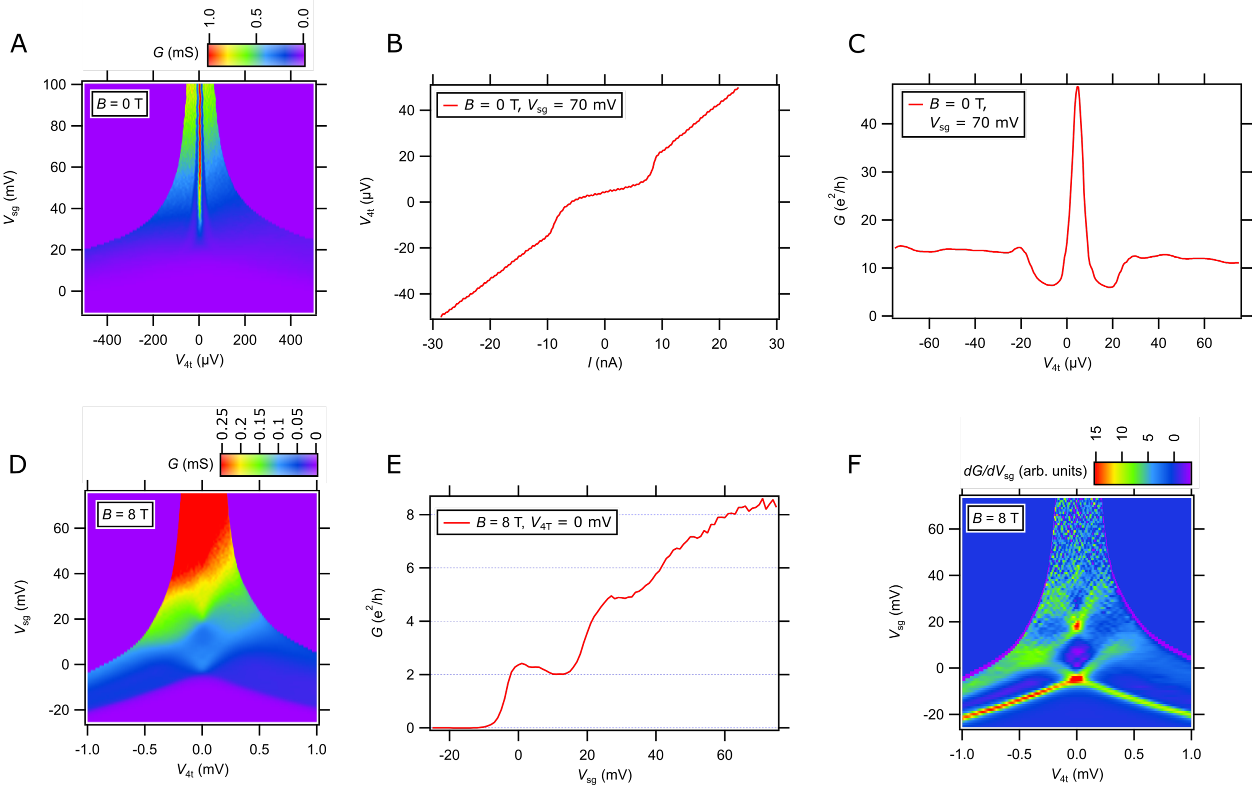

Figure S2: **Finite bias spectroscopy characterization of the superlattice section.** (A) IV curve spectroscopy for Device A at $B = 0$ T. (B) 4-terminal voltage as a function of current for the chiral superlattice section at $B = 0$ T and $V_{sg} = 70$ mV showing the superconducting state with a critical current of around 10 nA. (C) Horizontal linecut of the conductance map in (A) at $V_{sg} = 70$ mV showing the peak in conductance corresponding to the superconducting state. (D) IV curve spectroscopy at $B = 8$ T. (E) Vertical line cut at zero bias showing the conductance steps. (F) Transconductance showing characteristic diamond structure of ballistic transport through the superlattice device.

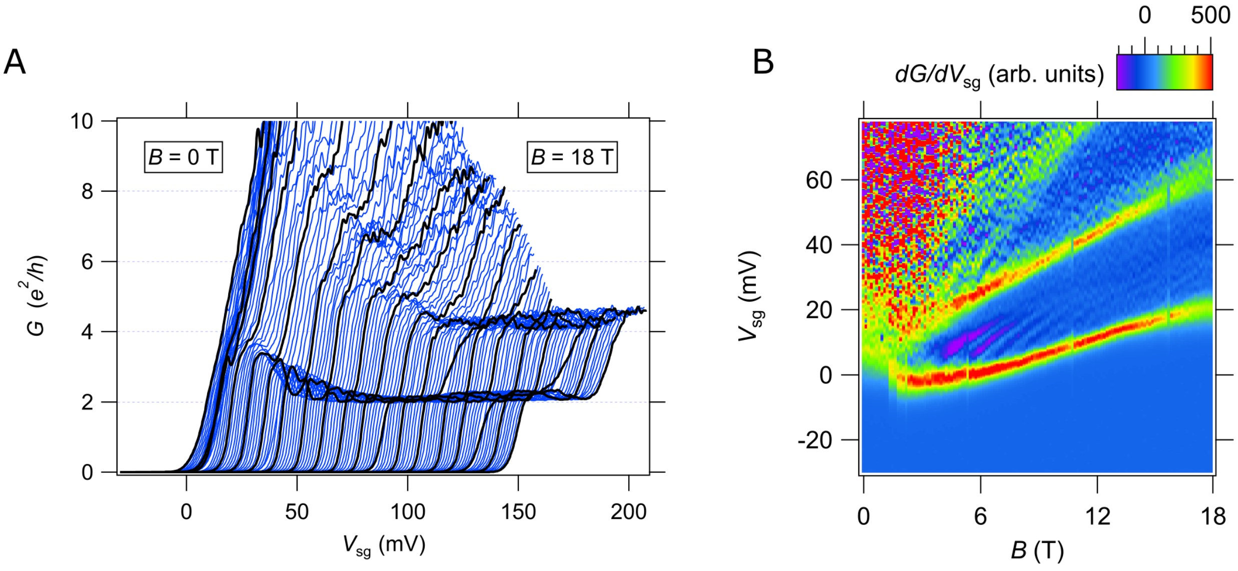

Figure S3: **Transport measurements under positive magnetic field conditions.** **(A)** Conductance curves for the superlattice section of Device A from 0 T to 18 T. **(B)** Transconductance dG/dV_{sg} map for positive magnetic field values. Transport for positive and negative applied magnetic fields is very similar. During this magnetic field sweep there were several spikes in the temperature of the dilution refrigerator causing distortions in the data at low field, and also periodically at around 5, 11, and 16 T.

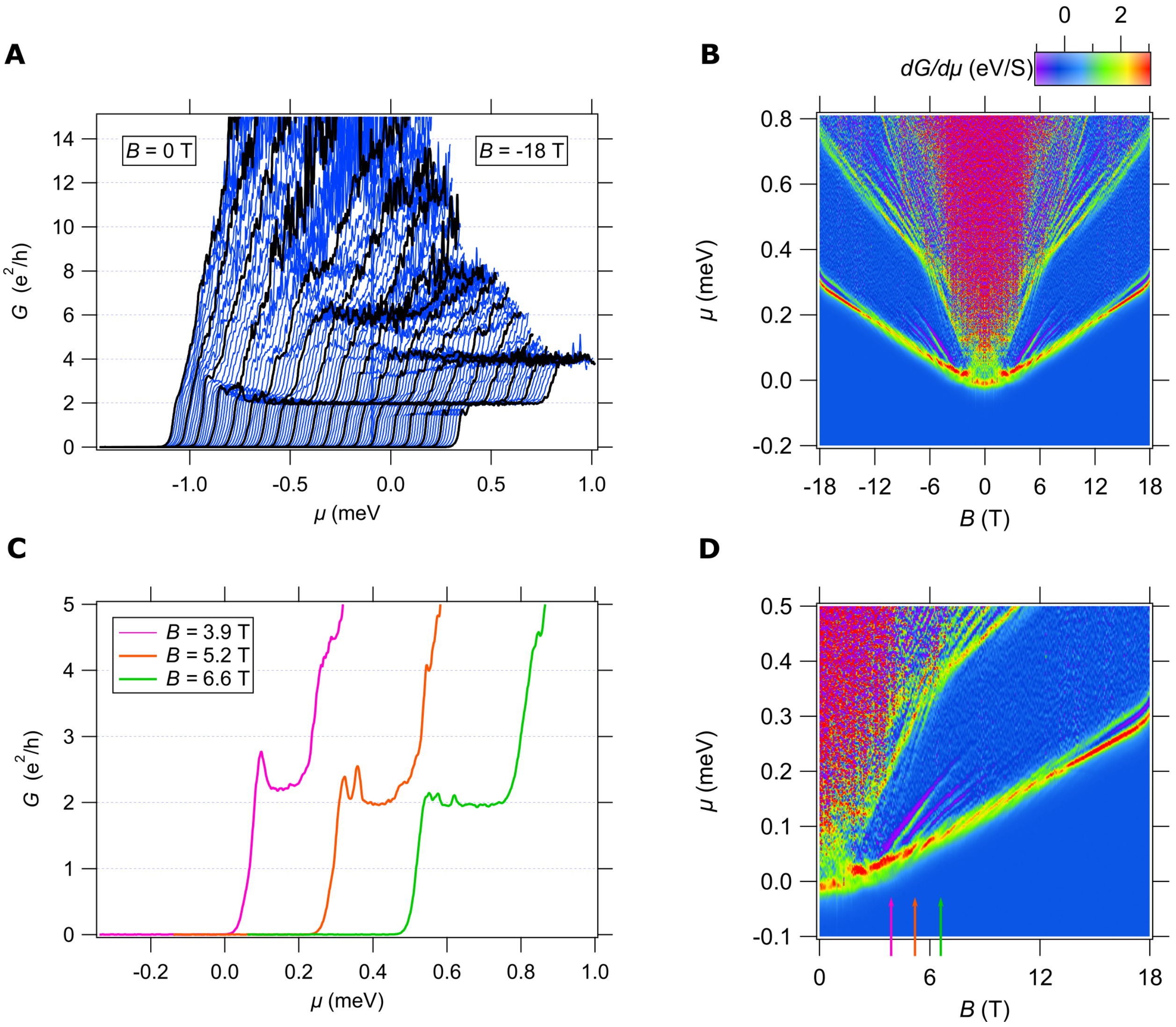

Figure S4: **Transport data from chiral superlattice Device B.** (A) Conductance data as a function of μ, curves are at different magnetic field values from $B = 0$ T to $B = -18$ T, curves are offset for clarity. The value of the first conductance plateau is at $G = 2e^2/h$ up to high magnetic field values, although there is a feature that appears at around $B = 15$ T. (B) Transconductance $dG/d\mu$ as a function of magnetic field B and chemical potential μ. Data is symmetrized. (C) Conductance line cuts at three magnetic field values showing the oscillations with a base conductance values of $G = 2e^2/h$. The number of oscillations increases with increasing B field. (D) Zoom in of the transconductance showing the oscillations and the lowest subband. Oscillations also appear in the lowest subband, where the conductance is increasing (bright regions) as well as on the plateau (dark regions).

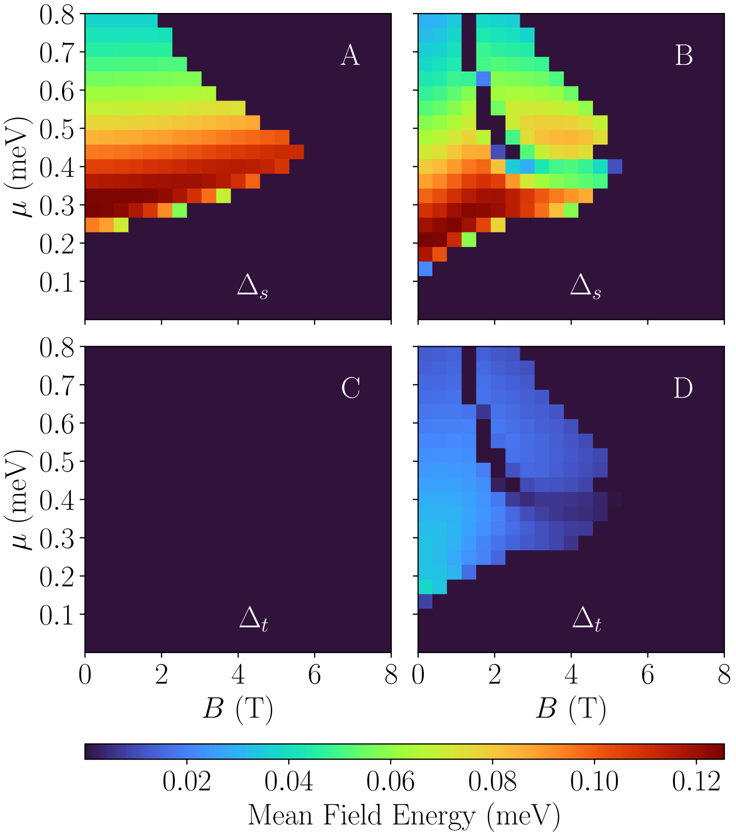

Figure S5: **Chiral waveguide potentials enable triplet pairing and enhance singlet pairing.** Singlet pairing fields $\Delta_s = \frac{U}{2\pi}\int \mathrm{d}k\ s_k$, with $s_k = \langle c_{-k,\downarrow} c_{k,\uparrow} - c_{-k,\uparrow} c_{k,\downarrow}\rangle/2$ are shown for the **(A)** straight, and **(B)** chiral waveguide in the top row, while the bottom row depicts the triplet pairing field $\Delta_t = \frac{U}{2\pi}\int \mathrm{d}k\ |t_k|$, with $t_k = \langle c_{-k,\downarrow} c_{k,\uparrow} + c_{-k,\uparrow} c_{k,\downarrow}\rangle/2$ in the **(C)** straight, and **(D)** chiral waveguide. Generally, a pairing field of $|\Delta| \gtrsim k_B T \approx 2.15\ \mu$eV indicates a paired phase associated with $2\frac{e^2}{h}$ conductance quanta. Here $c_{k,\sigma}$ is the fermionic annihilation operator for a subband electron of spin σ, and U is the attractive interaction strength. All simulations were performed for $m_x = m_y = 1.9 m_e$, $m_z = 6.5 m_e$, $g = 0.6$, $U_0 = -5.0$ meV·nm, $y_0 = 26$ nm, $z_0 = 8.1$ nm, and $T = 25$ mK. For the chiral waveguide we additionally used $A_y = \lambda = 10$ nm, $A_z = 0.2$ meV, and $\alpha_v = \alpha_l = 2.0$ meV·nm. The curved region of reduced pairing in the chiral waveguide is a direct result of the band gap introduced by the periodic modulation. Throughout, we have ensured the mean-fields are converged to a relative precision of 1%.

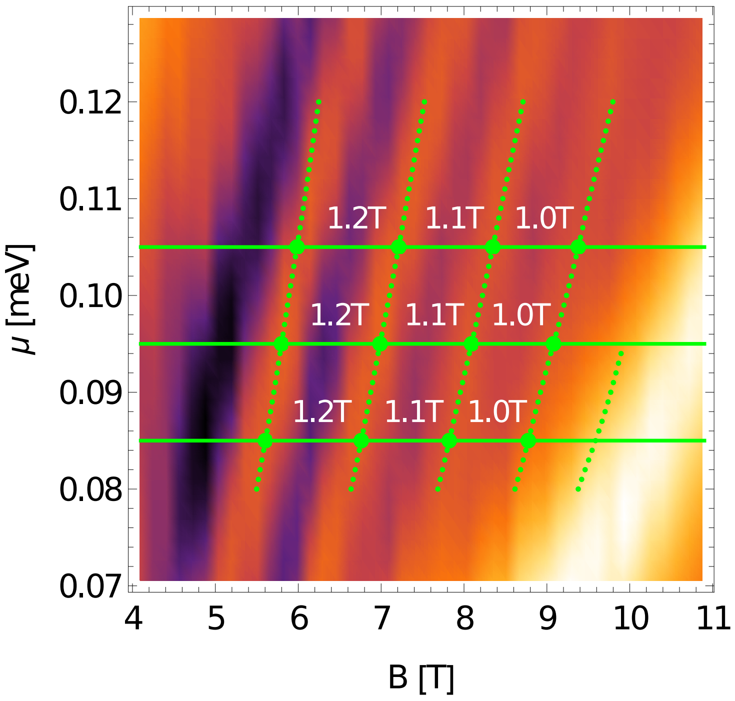

Figure S6: **Scattering model fits show good agreement with experimental transconductance patterns.** Fits to the experimental transconductance data. We have fitted the scattering model to the location of the fringes shown in Fig. 2. Maxima of the fitted scattering model are shown as green dots on top of the experimental data (contour background). The fitting parameters used are $\alpha = 0.45$ meVnm and $g = 0.85$. We find good qualitative agreement with the experimental data, and observe consistent magnetic field oscillation periods of $\Delta B \sim 1$T. These are shown in white between each fringe for three different chemical potentials.

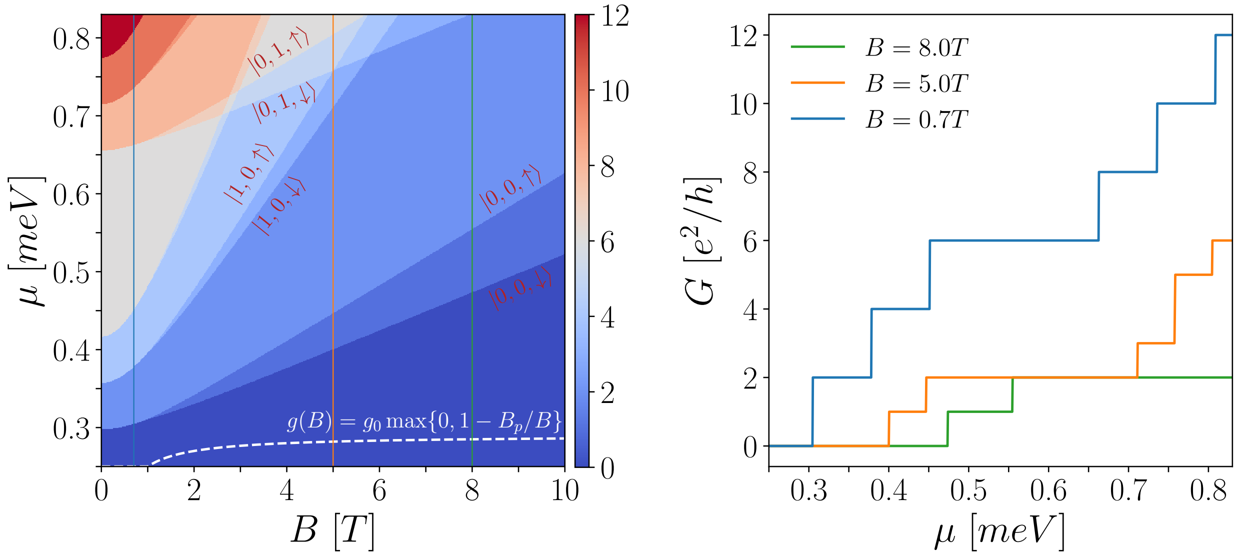

Figure S7: **Modelling the effect of renormalized g-factor on conductance behavior without spin-orbit coupling.** (left) Zero-bias conductance G (in units of e^2/h) as a function of magnetic field B and chemical potential μ for $\alpha_v = \alpha_l = 0.0$ meV nm. The dashed white line indicates the renormalized g-factor as a guide to the eye, while the three solid vertical lines show the values of B chosen to plot the conductance as a function of μ in three distinct regimes shown in the right panel. (right) Conductance as a function of μ for $B = 0.7$ T (blue), $B = 5$ T (orange) and $B = 8$ T. Other parameters are $m_x = m_y = 1.9m_e$ and $m_z = 6.5m_e$ where m_e is the electron mass, $l_y = \sqrt{\hbar/(m_y\omega_y)} = 26$ nm, and $l_z = \sqrt{\hbar/(m_z\omega_z)} = 8.1$ nm.

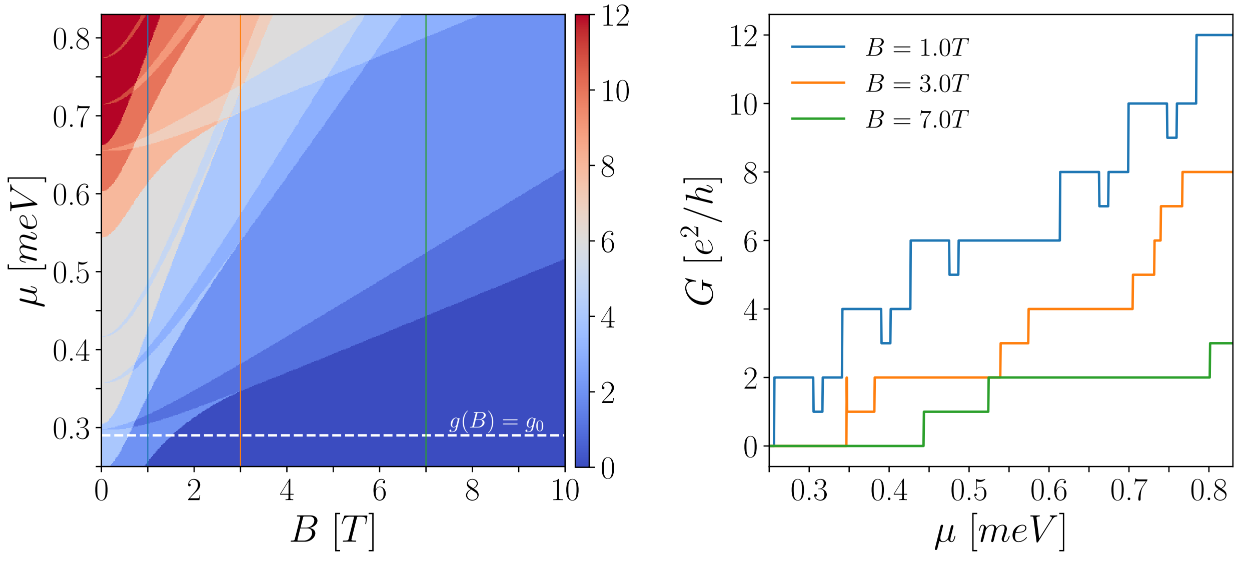

Figure S8: **Effect of vertical spin-orbit coupling on conductance demonstrates distinctive transport signatures.** (left) Zero-bias conductance G (in units of e^2/h) as a function of magnetic field B and chemical potential μ for $\alpha_v = 3.0$ meV nm, and $\alpha_l = 0.0$ meV nm. The dashed white line indicates the renormalized g-factor as a guide to the eye, while the three solid vertical lines show the values of B chosen to plot the conductance as a function of μ in three distinct regimes shown in the right panel. (right) Conductance as a function of μ for $B = 0.7$ T (blue), $B = 5$ T (orange) and $B = 8$ T. Other parameters are $m_x = m_y = 1.9m_e$ and $m_z = 6.5m_e$ where m_e is the electron mass, $l_y = \sqrt{\hbar/(m_y\omega_y)} = 26$ nm, and $l_z = \sqrt{\hbar/(m_z\omega_z)} = 8.1$ nm.

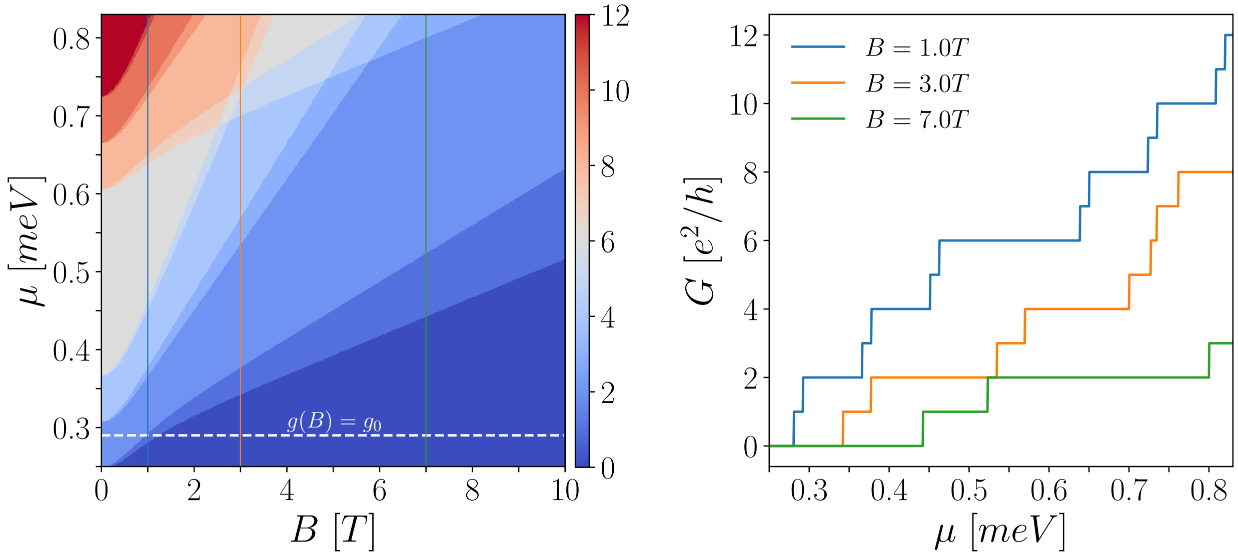

Figure S9: **Lateral spin-orbit coupling shows minimal influence on conductance structure compared to vertical coupling.** (left) Zero-bias conductance G (in units of e^2/h) as a function of magnetic field B and chemical potential μ for $\alpha_v = 0.0$ meV nm, and $\alpha_l = 2.0$ meV nm. The dashed white line indicates the renormalized g-factor as a guide to the eye, while the three solid vertical lines show the values of B chosen to plot the conductance as a function of μ in three distinct regimes shown in the right panel. (right) Conductance as a function of μ for $B = 0.7\,\mathrm{T}$ (blue), $B = 5\,\mathrm{T}$ (orange) and $B = 8\,\mathrm{T}$. Other parameters are $m_x = m_y = 1.9m_e$ and $m_z = 6.5m_e$ where m_e is the electron mass, $l_y = \sqrt{\hbar/(m_y\omega_y)} = 26$ nm, and $l_z = \sqrt{\hbar/(m_z\omega_z)} = 8.1$ nm.

Caption for Movie S1. Animated visualization of electronic density reveals helical trajectory in engineered chiral potentials. The supplemental video is an animated version of panels **A** and **B** of Figure 4, showing the electronic density and the trajectory of the center of mass of the electron along the waveguide in the first and second eigenstate of the chiral potential.
